# Zn/Fe remobilization from vegetative tissues to rice seeds: should I stay or should I go? Ask Zn/Fe supply!

**DOI:** 10.3389/fpls.2013.00464

**Published:** 2013-11-14

**Authors:** Raul A. Sperotto

**Affiliations:** Programa de Pós-Graduação em Biotecnologia, Centro de Ciências Biológicas e da Saúde, Centro Universitário UNIVATESLajeado, Brazil

**Keywords:** biofortification, metal remobilization, retranslocation, root uptake, xylem and phloem transport

Rice is a staple food for at least 50% of the world's population. Therefore, it is one of the most important crop plants on Earth (Lucca et al., 2002). However, milled rice is a poor source of essential micronutrients such as Fe and Zn (Bouis and Welch, 2010), whose deficiencies affect over three billion people worldwide, mostly in developing countries (Welch and Graham, 2004). Fe and Zn malnutrition are leading risk factors for disability and death, especially to children eating cereal-based diets, resulting in impaired functions of the brain, the immune and reproductive systems and energy metabolism (Graham et al., 2001). The development of new cultivars with elevated concentrations of Fe and Zn would be extremely relevant to alleviate malnutrition, but the lack of knowledge about how nutrients are translocated from vegetative tissues to the seeds is one of the barriers to rice biofortification (Colangelo and Guerinot, 2006; Sperotto et al., 2012a).

There are evidences in literature demonstrating that foliar applied Zn and Fe can be absorbed by leaf epidermis, remobilized, and transferred into the rice grains through the phloem (Wu et al., 2010; Wei et al., 2012; Yuan et al., 2012), presumably using several Zn- and Fe-regulated transporters (Schaaf et al., 2005; Bashir et al., 2012; Zhang et al., 2012). However, restrictions to the mobility of Zn and Fe supplied as cations can be expected due to the abundance of negative charges in the apoplastic space, which may limit their translocation to other plant compartments and/or organs (Fernández and Brown, 2013). Meanwhile, the translocation and redistribution of these minerals after its root uptake and passage to the transpiration stream have been extensively studied in the last years (for a comprehensive review see Ishimaru et al., 2011). Distribution of Fe and Zn within rice plants largely occurs through transport in the xylem, transfer from the xylem to the phloem, and retranslocation in the phloem (Ishimaru et al., 2011). Xylem transport is simply directed from roots to shoots in the transpiration stream, whereas phloem transport from old to new leaves is more selective, and is largely dependent on the phloem mobility of each element. In relation to their phloem mobility, Zn and Fe are considered intermediate or conditionally mobile (Fernández and Brown, 2013).

Remobilization of reserves to supply rice seeds with minerals has been emphasized in previous studies, but the contribution of stored minerals to total seed mineral content is unclear. During rice grain filling, Zn remobilization from leaves is not as important as Zn uptake by roots (Jiang et al., 2007). At the same time, increased root uptake does not necessarily result in enhanced Zn accumulation in rice grains (Jiang et al., 2008). None of the Zn foliar application treatments in rice showed that the main portion of Zn loaded in grain was remobilized from leaves (Jiang et al., 2008; Stomph et al., 2009). Wu et al. (2010) showed that large amounts of the Zn in rice seeds at maturity had been retranslocated from other plant parts and not directly acquired by the roots. Recently, Yoneyama et al. (2010) reported that Zn (and Fe) in the rice grains may be actively supplied via the phloem after mobilization from the leaf blades.

Iron stored in the flag and upper leaves may also be transported to the grains via the phloem. However, probably due to its limited mobility in the phloem, it seems difficult to improve the Fe nutrition of rice grain by Fe spray. Fang et al. (2008) were able to increase Fe content of rice grains by foliar application of Zn and Selenium. According to Yuan et al. (2012), after being taken up by the leaves, low-molecular-weight amino acids might chelate with Fe, which would increase the mobility of Fe and enhance its translocation to the sink during the development of rice grains. Grain Fe and Zn may share similar protein-dependent mechanisms for translocation to or storage in the grain, and several reports indicate a positive correlation between Fe and Zn grain concentrations (for a comprehensive review see Sperotto et al., 2012a). Such similarities between Fe and Zn raise the possibility of simultaneously biofortifying crops with more than one micronutrient, as previously found through the increase of nicotianamine concentration, a chelator of transition metals that plays important roles in long- and short-distance transport of metal cations (Johnson et al., 2011).

Flag leaf plays important roles in rice plants. It is already known that removal of the rice flag leaf at any stage after panicle emergence can cause significant reduction in grain yield (Abou-Khalifa et al., 2008), being the major component for yield losses due to the impaired synthesis and translocation of photoassimilates. On the other hand, it seems that minerals do not have the same behavior of photoassimilates, since no single report has pointed flag leaves as the major source of Fe and Zn to the rice developing seeds. As it is hypothesized that flag leaves could have a role in Fe and Zn remobilization to rice seeds, several reports tried to find a relationship between gene expression in flag leaves with concentration of mineral nutrients in rice seeds (Narayanan et al., 2007; Sperotto et al., 2009, 2010; Zhang et al., 2012).

To address this question, Sperotto et al. (2013) conducted field experiments to evaluate the effect of flag leaf removal (at anthesis) on seed Fe and Zn concentration and content. Seed Fe and Zn accumulation were not affected by flag leaf removal, suggesting that the flag leaf is not absolutely required for metal remobilization to the seeds of rice plants growing in field conditions. These authors also removed the second upper leaf and found similar results. Probably, the lack of flag leaves is compensated by other leaves and stem/sheath remobilization and/or continuous uptake by roots. In *Arabidopsis*, continuous uptake and translocation of minerals to source tissues during seed fill is as important, if not more important, than remobilization of previously stored minerals (Waters and Grusak, 2008).

In another experiment, Sperotto et al. (2012b) showed that mineral remobilization from green tissues can be severely affected by Fe status. Rice plants watered with a high Fe concentration showed no Fe remobilization from flag leaves, non-flag leaves and stems/sheaths. On the other hand, plants watered with low Fe concentration showed the highest Fe remobilization from stems/sheaths, probably due to reduced uptake from the roots during seed fill. Plants watered with a sufficient Fe concentration showed high levels of mineral remobilization (including Fe and Zn) mostly from flag leaves but also from stems/sheaths. However, as the flag leaves mineral content is much lower than the stems/sheaths content, the maximum possible contribution to seed mineral content is, in general, higher from stems/sheaths than from flag leaves (Sperotto et al., 2012b). It seems that abundant Fe supply at the root level promotes continued uptake during seed fill, which may have reduced the need for remobilization to serve as a source of Fe for seeds. A similar pattern was observed for Zn (Jiang et al., 2007, 2008; Impa et al., 2013). According to Jiang et al. (2007), in rice plants grown under sufficient or surplus Zn supply, most of the Zn accumulated in the grain originates from uptake by roots after flowering and not from Zn remobilization from leaves. It was also shown that, at lower Zn supply levels, the Zn taken up by the roots after flowering seems to accumulate mostly in the grain, which is accompanied by net Zn remobilization from the leaves and transport to the grain. However, at higher Zn supply levels, Zn content in all non-grain organs remained constant (roots, leaves and sheaths) or continued to increase after flowering, and grain Zn accumulation could be fully accounted for by Zn uptake during grain filling (Jiang et al., 2008).

Evidences show that Zn and Fe remobilization from vegetative tissues can occur in rice plants; however, remobilization is not required for seeds to acquire minerals. Contrasting results may be due to different rice genotypes behaving differently. Thus, there may not be only one way for rice to load Zn/Fe into grain, but genotype-specific variations. Such useful variations should be used as targets for biofortification breeding strategies. Probably, the xylem discontinuity at the base of the rice grain (Stomph et al., 2009) can contribute to the fact that continued uptake and translocation of minerals to source tissues during seed fill is as or more important than remobilization of previously stored minerals from vegetative tissues. Remobilization results found for *Arabidopsis* plants by Waters and Grusak (2008) were not exactly consistent between experiments, as differences were found in elements and in amounts remobilized. Also, mineral remobilization from vegetative tissues in rice seems to be modified by plant Zn and Fe nutrition, because different Zn/Fe supplies alter the remobilization levels. Probably, rice genotypes with different efficiencies in the use of Zn and Fe and also with different levels of Zn and Fe in the seeds can show different remobilization patterns, since mineral transport can be influenced by minimal changes in source-sink communication (Jiang et al., 2008; Wu et al., 2010; Impa et al., 2013).

**Figure 1:**
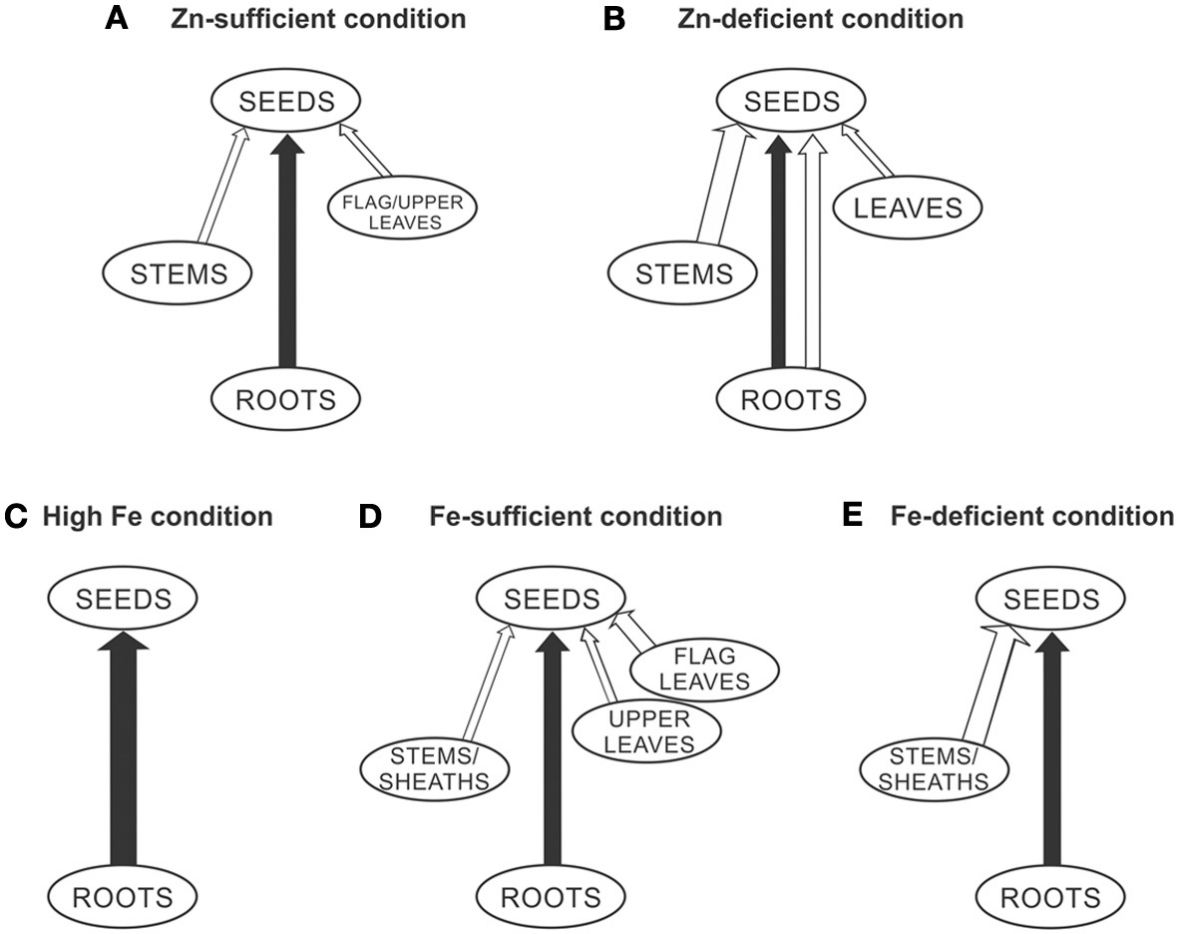
**Proposed model for Zn and Fe allocation to the rice seeds under different Zn and Fe supplies (A, Zn-sufficient condition; B, Zn-deficient condition; C, High Fe condition; D, Fe-sufficient condition; E, Fe-deficient condition), through the xylem (continued root uptake, black lines) and through the phloem (mineral remobilization, white lines)**. The thicknesses of the lines represent more or less active processes.

Based on the findings of different studies (Jiang et al., 2007, 2008; Wu et al., 2010; Yoneyama et al., 2010; Sperotto et al., 2012b; Impa et al., 2013), a proposed model for Zn and Fe allocation to the rice grains is shown in Figure 1. Under Zn-sufficient conditions (Figure 1A), Zn in the rice grain is mostly supplied by continued root uptake during grain-filling stage. To a lesser extent, Zn from stems and flag/upper leaves can be remobilized. Under low Zn supply (Figure 1B), both continued root uptake and also remobilization from roots, stems, and leaves can occur. It is important to note that high Zn availability is rarely found in field conditions, even with adequate fertilizer application, being most common in artificial growth, under laboratory conditions. Under high Fe conditions (Figure 1C), continued root uptake can fully account for seed Fe allocation. Under control (or sufficient) Fe supply (Figure 1D), continued root uptake and also remobilization from stems/sheaths and flag/upper leaves contribute to total seed Fe. Under Fe-deficient conditions (Figure 1E), seed Fe can be fully addressed by remobilization from stems/sheaths and continued root uptake. It is important to highlight that this model only summarize previous studies, and cannot be used *per se* to help rice researchers to increase seed mineral concentrations. For this purpose, we would have to discover how to “trick” rice plants through manipulation of transport and accumulation processes, in order to efficiently increase Zn and Fe concentrations into grains.

Loading and bioavailability of minerals in the rice grains, mainly in milled rice, not only follows the transport and remobilization pathway. It also depends on the mechanism of entry from grain aleurone layer to the inner endosperm. Several barriers to Fe entry (and probably to Zn) were identified, as transport proteins which only internalize Fe during germination, chelating molecules such as nicotianamine and deoximugineic acid, low levels of ferritin protein in the endosperm tissue and mostly high levels of phytic acid (Paul et al., 2013), which is a potent inhibitor of Fe absorption (also called anti-nutrient factor). Phytic acid localizes almost exclusively to the bran of brown rice (Prom-u-thai et al., 2008), acting as a barrier to endosperm internalization. As biofortification efforts should focus primarily on increasing endosperm Zn/Fe bioavailability, reductions of phytic acid content would be important for rice biofortification, and several promising biotechnological approaches have been studied. Anyway, understanding the mechanisms of Zn and Fe remobilization from vegetative tissues and seed loading and bioavailability in rice is crucial to the enrichment of rice with Zn and Fe, which is a way to generate major health benefits for a large number of susceptible people.
